# An evidence-based method for assessing the value of a search tool: a pilot study

**DOI:** 10.5195/jmla.2018.287

**Published:** 2018-10-01

**Authors:** Donald Stanley Pearson, Stevo Roksandic, Jill Kilanowski

**Affiliations:** Manager, Patient and Family Resource Center, James Cancer Hospital and Solove Research Institute, The Ohio State University Comprehensive Cancer Center, Room C001, Ground Floor Lobby, 460 West 10th Avenue, Columbus, OH 43210; Regional Director, Library Services, Mount Carmel Health System, 127 South Davis Avenue, Columbus, OH 43222; Associate Dean, Graduate Program, and Professor, Mount Carmel College of Nursing, Columbus, OH

## Abstract

**Objective:**

The objective of this study was to develop an evidence-based method with a set of metrics that could be used to assess an information search tool.

**Methods:**

This pilot study analyzed a two-group convenience sample of graduate nursing students and resident physicians. The intervention group received ten minutes of instruction on a familiar search tool (eSearcher). Each group was provided one prompt to search for clinical guidelines on a given topic within their scope of practice and asked to find the best result using only eSearcher (intervention group) or specifically excluding eSearcher (comparison group). Three measurements of search results were employed: time elapsed to complete the search, an accuracy score, and a participant-reported score of confidence in the result.

**Results:**

Forty-two students participated in this study (23 graduate nursing students and 19 resident physicians). The intervention group consisted of 22 participants (12 graduate nursing students and 10 resident physicians), and the comparison group consisted of 20 participants (11 graduate nursing students and 9 resident physicians). The intervention group had lower mean ranks in both accuracy and confidence compared to the comparison (not eSearcher) group, although these differences were not significant. However, the intervention (eSearcher) group had significantly longer search times compared to the comparison (not eSearcher) group.

**Discussion:**

These findings provided new insights into the performance of the search tool and how users felt about their search experience. The quantitative evidence gained from this study led directly to an informed decision to explore other options for search tools. The evidence-based methods and process developed in this pilot study will enable similar studies to test other student groups and other search tools, leading to better informed purchasing and instructional decisions.

## INTRODUCTION

An important aspect of managing library digital resources is determining their usefulness and value to library users. Resources employed in medical libraries have an even stricter mandate to provide the most up-to-date, peer-reviewed content, often with an additional time constraint for rush patient care. This perceived need has opened up another line item in many medical libraries’ budgets: a multi-search discovery product that provides simultaneous access to multiple types of materials, independently of the management platform involved [1]. Discovery products provide an interface with search and retrieval capabilities, often with advanced filtering and sharing features. Medical librarians and their clients need the sophisticated searching tools of resources like PubMed but also insist on the speed and simplicity of search engines like Google. Finding such a high-performance tool that searches medical information is a difficult task in collection development for electronic resources.

In 2012, in response to this need for quick and thorough medical database searches, Mount Carmel Health Sciences Library (MCHSL) implemented a unique federated search tool that specialized in simultaneously searching the library’s suite of 24 biomedical databases, including PubMed and products from Elsevier (ClinicalKey), McGraw-Hill (AccessMedicine), ProQuest, and EBSCO, as well as the library’s online catalog of electronic books and journals. For marketing purposes, the search tool was branded as eSearcher. Over four years, eSearcher performed satisfactorily with users commenting on its speed in returning relevant results, visual appeal, filters, and overall simplicity and ease of use. Usage statistics indicated a steady increase of queries: rising from around 500 monthly queries in October 2012 to more than 1,500 monthly queries in April 2016. During this time, librarians heavily promoted the use of eSearcher in library orientations and other instruction sessions.

After four years of use, MCHSL management wanted to assess the usability and performance of this new tool. The only quantitative assessment data were usage counter statistics (COUNTER), which were provided directly from the eSearcher vendor. These usage COUNTER statistics were supplemented with anecdotal accounts of resource usefulness from Mount Carmel Health System Graduate Medical Education and Mount Carmel College of Nursing faculty, staff, and students. Voluntary, online surveys were also used occasionally, but neither method was ever performed in a systematic, comparative way. Therefore, MCHSL management felt a need to develop a more quantitative and evidence-based method of assessing the usefulness of an online search tool.

The objective of this study was to develop an evidence-based method of assessing the accuracy of and users’ confidence in using an information search tool that could be general enough to be employed in any user population with any online search tool, whether it be a single database, a federated search, a discovery layer, or another tool developed in the future. The study’s question was: “How can we easily and quickly evaluate the performance of a search tool from a user’s perspective?” The immediate goal of the pilot study was to gather data that would allow a more careful assessment of eSearcher’s performance and usefulness as a primary search interface through engaging postbaccalaureate trainees in a simulated topic search.

## METHODS

This study specifically targeted postbaccalaureate health care trainees (graduate nursing students and resident physicians) as they searched for evidence-based clinical practice guidelines that were typically found in the National Guideline Clearinghouse. Clinical practice guidelines are systematic reviews of evidence that offer an evaluation of the quality of the relevant scientific literature and include an assessment of the benefits and harms of alternative care options [2]. Clinical practice guidelines assist health care practitioners in selecting the best care for an individual patient based on his or her preferences [2]. Providing access to clinical guidelines is an important service that the library offers to patrons. To that end, the library has sought to both train students to be information literate and provide tools to streamline the search process.

To develop the methodology for this pilot study, the investigators reviewed previous evidence-based studies of search tools, paying special attention to those involving the search for medical information. Georgas conducted sessions where participants were divided up into 2 groups, with one group asked to use a specific search tool only, and the other asked to use Google. Our study utilized her 3 distinct but interrelated measures of users’ search experience: searching habits (which we distilled to time), analysis of results, and the user’s own perceptions [3]. Belliston et al. similarly sought to gather 4 data points on each search, including time, the accuracy of the results, user satisfaction, and preference [4]. The measure in our study was a simplified version that used a single Likert scale of 0 to 7 to elicit a confidence score. Our study was modeled most closely after Thiele et al.’s study, which focused on the ability of the search tools to answer clinical questions, in terms of 3 metrics: accuracy, speed (within an allotted 5-minute period), and user confidence [5].

To address concern about the influence that the novelty of the search tool might have on the study, Fagan et al. [6] and Comeaux [7] employed a pretest questionnaire in order to gauge the extent of users’ general experience of, training on, and familiarity with the search tool. Following their example, in the months before the search activity, we conducted a SurveyMonkey® survey that helped to gauge the study’s user population’s general familiarity with eSearcher (supplemental Appendix A). We also followed the practice of Fagan et al. [6] by allowing some free exploration time with eSearcher before the actual study task to further diminish the effect of the novelty of the tool itself on search time.

The MCHS Institutional Review Board (IRB) exempted the study from review due to its minimal risk to subjects. We obtained an email list from the program registrars of the target population, including 153 graduate nursing students and 80 resident physicians. Ultimately, 42 individuals participated in the study. Participants were informed of the study requirements and consented before active engagement in the activity.

The thirty-minute search activity took place in a closed computer lab with two health sciences librarians present. Participants were divided into two groups using alternate placement. Paper response forms were distributed to both groups of participants before the search activity (supplemental Appendix B). The forms included written directions for the search prompt. For resident physicians, the prompt was to find guidelines on the administration of prophylactic antibiotics to a pregnant patient. For graduate nursing students, the prompt was to find guidelines on the administration of medications in managing asthma. These prompts were selected after guidance from faculty on a topic that was within the scope of practice for each trainee type. There were spaces on the response form to enter basic demographic information, the start and stop times of the search, the answer to the search prompt, a Likert-type scale for recording confidence level, and a free space to record any comments.

Before the search activity, all participants were read the same script about the study and its purpose. Participants in the intervention group then received ten minutes of instruction on eSearcher from a health sciences librarian and were allowed another five minutes to freely explore the tool before the activity began. Participants in the comparison group received no instruction or exploration time. To complete the search activity, the intervention group was asked to use only eSearcher, and the comparison group was asked to use any online resource except eSearcher. At the conclusion of the search activity, participants were asked to leave their screens open to their final answers. The investigators printed the screen of each computer and affixed the printout to that participant’s response form.

Three scores were recorded for each participant: (1) time (in minutes) to complete the search, (2) an accuracy score, and (3) the participant’s rating of confidence in their answer. The total time for the search was calculated by subtracting start from stop time as recorded on the response form.

Accuracy was determined by a 4-person panel including 2 health sciences librarians, 1 library assistant who is also a registered nurse, and 1 library technology specialist. Each panelist independently graded each participant’s search result to score the accuracy of the search. The panel used both the participant’s stated answer on the response form and the printout of the final search screen to ascertain the intended final answer. The scoring rubric ranged from 0 to 3, depending on the number of criteria the answer met (0=no criteria met, 1=1–2 criteria met, 2=3 criteria met, 3=all criteria met). The four criteria were that the answer (1) was a guideline, (2) was the most current guideline, (3) addressed the correct patient population, and (4) was authored in the United States. Following individual grading, the panel met to reach a consensus to award a final grade for accuracy.

The confidence score was self-reported by participants using a Likert-type scale with a range of 0 to 7 (from “not at all confident” to “extremely confident”) with 4 being the neutral or undecided option. Finally, all data from the response forms were transcribed into a single Microsoft Excel spreadsheet that was then imported into SPSS for statistical analyses. Due to our small sample size and non-normal distribution of the data, the data were analyzed using non-parametric Mann-Whitney U tests to compare differences between resident physicians and graduate nursing students.

## RESULTS

Of the 80 resident physicians in our programs, 19 participated in our search activity (24% response rate), and of 153 graduate nursing students, 23 participated in the search activity (15% response rate). There were 26 women and 15 men in the study, with 1 person not identifying gender. The largest participant age group was 26 to 30 years (31%), followed by the age group of 31 to 35 years (24%).

The time to complete the search ranged from 1 minute to 16 minutes, with a mean time to completion of 6 minutes. Accuracy scores ranged from 0 to 3, with a mean score of 1.79. Errors in accuracy were broken down as follows: 48% selected resources that were not guidelines, 38% did not select the most recent guideline, 31% selected a guideline for an incorrect patient population, and 24% selected a guideline authored outside the United States. Participant confidence scores ranged from 2 to 7, with a mean confidence score of 5.60.

Compared with participants in the comparison group (not using eSearcher), participants in the intervention group (eSearcher) had longer search times, lower mean accuracy scores, and were less confident of their answers (Table 1). The most confident searchers (mean confidence level of 5.85) were those who had gotten only 2 out of 4 accuracy categories correct (i.e., score of 1 out of 3). This “overconfident” group represented 31% of our study participants (13 out of 42 individuals).

**Table 1:** Study results

Participants	Sample size	Time				Accuracy				Confidence			

		Average time to complete the search activity in minutes with standard deviation				Average score by panel using rubric * with standard deviation				Average confidence level as reported by participants† with standard deviation			

		eSearcher	(SD)	Not eSearcher	(SD)	eSearcher	(SD)	Not eSearcher	(SD)	eSearcher	(SD)	Not eSearcher	(SD)
Graduate nursing students	23 (12 eSearcher, 11 not eSearcher)	8.08	(3.58)	5.00	(1.94)	1.25	(1.06)	1.46	(0.82)	4.92	(1.44)	6.09	(0.83)
Resident physicians	19 (10 eSearcher, 9 not eSearcher)	5.33	(2.78)	4.13	(3.18)	2.20	(1.14)	2.44	(1.13)	5.70	(0.82)	5.78	(1.09)
All participants	42	6.90	(3.38)	4.61	(2.52)	1.68	(1.17)	1.90	(1.07)	5.27	(1.24)	5.95	(0.95)

* Higher score=greater accuracy.

† Higher score equals greater confidence.

Mann-Whitney U tests were used to compare differences between the intervention (eSearcher) and comparison (not eSearcher) groups. The intervention (eSearcher) group had lower mean ranks in both accuracy and confidence compared to the comparison (not eSearcher) group, although these differences were not significant. However, the intervention (eSearcher) group had a significantly higher mean rank in search time compared to the comparison (not eSearcher) group (U=107; n=39; *p*=0.027).

In the 2 professional categories of our sample, resident physicians had higher accuracy and confidence mean rank scores compared to graduate nursing students. However, only accuracy scores were significantly different between groups (U=109.5; n=42; *p*=0.004). Resident physicians had lower search times compared to graduate nursing students, but this difference was not significant.

There were no significant associations among the 3 study metrics as evaluated by the Spearman’s *rho* test. Due to limitations of study design, individual accuracy scoring from the 4-person panel was only saved for the graduate nurse participants, for which inter-rater reliability had a Cronbach’s alpha of 0.900.

## DISCUSSION

Based on previous studies by Belliston et al. [4], Thiele et al. [5], and Georgas [3], we employed an easily administered yet reasonably diversified method to assess a search tool. Given the fast change in technology and the busy schedules of the library’s medical professional clients, we sought to develop a quick but thorough method to analyze search accuracy and user attitudes and perceptions. We now have a way to gauge the time, accuracy, and confidence level of a search tool used by any user group.

The data gathered by this pilot study answered the research question: “How can we easily and quickly evaluate the performance of a search tool from a user’s perspective?” A procedure was developed that gave us a way to assess our search tool using the concrete metrics of time, accuracy, and confidence level. Although the librarians received anecdotal positive comments about eSearcher from our library users and its usage statistics increased over time, the study findings showed that eSearcher did not add value to our users’ search process in any of the three measurements. Participants in the intervention group, who used eSearcher, spent significantly more time performing the search than participants in the comparison group. Moreover, the use of eSearcher did not result in better accuracy and confidence scores than the use of other tools and search strategies. Of note, the significantly higher accuracy scores of resident physicians compared with graduate nursing students could be explained by their four additional years of postbaccalaureate education and more experience in using search engines.

We took several measures to ensure that the intervention tool (eSearcher) was familiar to users. A SurveyMonkey survey was distributed to library users a short time before this study, which revealed that 65% of respondents reported at least some degree of familiarity with the tool. Additionally, eSearcher had been in use by our students for 4 years previous to the study. It was highlighted at the top center of all pages of the library website, and it had been actively promoted in library orientations, instruction, and other communications. Four years of usage statistics showed a 3-fold rise in use over 4 years, during which the eSearcher tool received more promotion and instruction than any other single resource in the library. Because of this, we do not think that any novelty of the tool had a great effect on the measurement of search time or users’ confidence level.

Study limitations include a small sample size, the lack of a pre- and post-test knowledge assessment, incomplete labeling of the Likert scale of confidence, and incomplete grading records. We did not record each individual rater’s scores for the resident physicians’ search results and, thus, were unable to calculate inter-rater reliability for this group of participants. However, the raters’ accuracy scores for graduate nursing students were recorded and preserved. These mistakes are acknowledged in this pilot study and will influence the design of subsequent studies.

In the future, the sample size of the study may be increased through refined recruitment efforts and possible incentives for participation. Although this pilot study analyzed a federated search tool that postbaccalaureate students use, future studies may use the same methodology to assess other search tools, such as those that undergraduates use. The addition of a pre- and post-test survey given during the actual laboratory exercise would better reflect any effect of the instructional intervention. Also, the Likert-type question should be modified to include full labels of the ordinal scale, not just the first and last values, with the center or neutral choice also clearly labeled. With regard to accuracy grading, we would modify the grading scale to be 0 to 4 instead of 0 to 3, in order to more clearly reflect how many criteria were satisfied. Future studies will preserve all scores for all raters so that complete inter-rater reliability can be computed. In addition, training sessions for the scoring panel will include practice on accuracy scoring in adherence to a written rubric.

The study findings echo the results of Krause et al., which suggest that confidence does not always correspond to accuracy when it comes to online searching. Krause et al. found in their study of emergency medicine residents’ use of Google that “unsure answers decreased, whereas incorrect answers increased” [8]. This study suggests that this effect might not be unique to Google and reinforces our impression of the need for more information literacy instruction rather than more sophisticated search tools. Indeed, Krause et al. conclude their study with the suggestion that, “Enlisting the assistance of a health sciences librarian in providing search-strategy training to residents, medical students, and attending physicians can overcome many of the associated pitfalls [of using technologic innovations]” [8].

The study results allowed our library management to make an informed decision to explore other search tools that were already available via an existing consortium package, effectively removing this expense item from the library budget. Our study data also suggested that there was not an overwhelmingly positive value to having an additional multi-search tool at this time, which led the investigators to speculate that the time spent searching for and implementing a more advanced search tool might be better spent on developing better information literacy programs and instruction.

This pilot study offers a way to quantitatively assess a search tool for timeliness, accuracy, and user confidence. Other libraries may use this methodology for other search tools as well as student populations in other academic disciplines. Although no search tool is a replacement for solid information literacy instruction, this study developed an evidence-based method to determine the value of a search tool and make an informed purchasing decision.

## SUPPLEMENTAL FILES

Appendix A eSearcher survey results

Appendix B eSearcher study paper response form
