# Supplementary material for: An evidence-based method for assessing the value of a search tool: a pilot study
Source: J Med Libr Assoc. 2018 Oct 1;106(4):471–6. doi: 10.5195/jmla.2018.287 (PMC6148619; doi:10.5195/jmla.2018.287)
Supplement: Appendix A [file jmla-106-471-s001.pdf]

JMLA Journal of the Medical Library Association

Supplemental content to
J Med Libr Assoc. Oct;106(4):dx.doi.org/10.5195/jmla.2018.287
www.jmla.mlanet.org
© Pearson, Roksandic, Kilanowski 2018

An evidence-based method for assessing the value of a search tool: a pilot study

Donald Stanley Pearson, MBA, MLIS, AHIP; Stevo Roksandic, MBA, MLIS, AHIP; Jill Kilanowski, PhD, RN, APRN, CPNP, FAAN

APPENDIX A

eSearcher survey results

83 total responses

1. How often do you use the Internet?

Answer	# responses	Total number	Percent
Daily	80	82	97.56
Weekly	2	82	2.44
Monthly	0	82	0

2. What is your age group?

Answer	# responses	Total number	Percent
26–30	27	82	32.93
31–35	20	82	24.39
50>	16	82	19.51
41–45	8	82	9.76
36–40	4	82	4.88
46–50	4	82	4.88
21–25	3	82	3.66
<20	0	82	0

3. What is your gender?

Answer	#responses	Total number	Percent
Female	52	83	62.65
Male	31	83	37.35

4. I visit the Mount Carmel Health System (MCHS) Library website:

Answer	# responses	Total number	Percent
Several times a week	33	83	39.76
Weekly	17	83	20.48
Monthly	16	83	19.28
Rarely	9	83	10.84
Daily	8	83	9.64

JMLA Journal of the Medical Library Association

SC

Supplemental content to
J Med Libr Assoc. Oct;106(4):dx.doi.org/10.5195/jmla.2018.287
www.jmla.mlanet.org
© Pearson, Roksandic, Kilanowski 2018

5. I access the MCHS Library most often:

Answer	# responses	Total number	Percent
From home	42	83	50.60
At work	29	83	34.94
Remotely using a portable device	8	83	9.64
In the MCHS Library	3	83	3.61
Other	1	83	1.20

6. I visit MCHSL website because I need (check all that apply):

Answer	# responses	Percent
To find articles	70	84.34
To find information for research	66	79.52
To find information for clinical patient care	53	63.86
To find information for school/self-education	53	63.86
To find/use electronic resources such as databases, e-journals, e-books, etc.	50	60.24
To locate Policies & Procedures and/or Standards & Guidelines	17	20.48
To locate information about the library, read news, look for new library materials	4	4.82
Other	2	2.41

7. In which MCHS graduate education program are you enrolled?

Answer	# responses	Total number	Percent
Graduate nursing	40	76	52.63
Graduate medical	36	76	47.37
Skipped	7	83	

8. If you are enrolled in graduate nursing education program, please choose the program you are enrolled in and how many credit hours have you completed?

Answer	<10	11–20	21–30	31>	Total
Adult-Gero CSN	0	0	5	0	5
Adult-Gero Acute Care NP	1	0	1	1	3
Family NP	3	4	1	4	12
Nursing administration	1	2	2	4	9
Nursing education	3	2	6	3	14
Total	8	8	15	12	43
Skipped	40 out of 83				

Journal of the Medical Library Association

Supplemental content to
J Med Libr Assoc. Oct;106(4):dx.doi.org/10.5195/jmla.2018.287
www.jmla.mlanet.org
© Pearson, Roksandic, Kilanowski 2018

9. If you are enrolled in Graduate Medical Education Residency Program, please choose the program you are enrolled in and what year of residency you are in.

Answer	PGY-1	PGY-2	PGY-3	PGY-4	PGY-5	PGY-6	Total
Family medicine	2	1	1	1	0	0	5
Internal medicine	7	3	3	1	0	0	14
Orthopedic	2	1	1	0	1	0	5
Surgery	2	1	0	1	1	1	6
Transitional year	4	0	0	0	0	0	4
OB/GYN	0	0	0	1	0	0	1
Colon and rectal surgery	0	0	0	0	0	0	0
Total	17	6	5	4	2	1	35
Skipped	49 out of 83						

10. How often do you use Google to find clinical information?

Answer	# responses	Percent
Daily	35	42.17
Weekly	24	28.92
When I do not find answers anywhere else	15	18.07
Never	5	6.02
Monthly	4	4.82

11. Where do you prefer to search for clinical information?

Answer	# responses	Percent
MCHS Library site	54	71.05
Google	22	28.95
Skipped	7 of 83	8.43

SC
Supplemental content to
J Med Libr Assoc. Oct;106(4):dx.doi.org/10.5195/jmla.2018.287
www.jmla.mlanet.org
© Pearson, Roksandic, Kilanowski 2018

12. Please choose your preferred database used to search for clinical information for your medical education:

Answer	# responses	Percent
UpToDate	29	34.94
EBSCO Nursing Databases	16	19.28
CINAHL	15	18.07
PubMed	11	13.25
MCHS Library eSearcher	8	9.64
Clinical Key	3	3.61
Lippincott Nursing Resources (Advisor, Procedures)	1	1.20
Cochrane Library	0	0

13. Please share briefly why do you prefer this database resource over the Google option? (65 text responses)

---

14. How familiar are you with MCHS Library eSearcher?

Answer	# responses	Percent
Completely unfamiliar	28	34.57
Somewhat familiar	24	29.63
Familiar	23	28.40
Very familiar	6	7.41
Skipped	2	2.40

15. If you are familiar with eSearcher, how often do you use this MCHSL eSearcher?

Answer	# responses	Percent
Occasionally	22	32.35
Never	18	26.47
Often	18	26.47
Not sure	8	11.76
Very often	2	2.94
Skipped	15 of 83	18.07

Supplemental content to
J Med Libr Assoc. Oct;106(4):dx.doi.org/10.5195/jmla.2018.287
www.jmla.mlanet.org
© Pearson, Roksandic, Kilanowski 2018

16. If you are somewhat familiar, familiar, or very familiar with eSearcher, please rate the quality of eSearcher on the following factors:

Answer	Poor	Fair	Good	Excellent	N/A	Total	Wt. Avg.
Comprehensiveness, breadth, and depth	1	10	27	15	8	61	3.06
Clear and understandable language	1	10	28	13	8	60	3.02
Usefulness of information	1	12	27	13	8	61	2.98
General appearance	1	11	31	10	8	61	2.94
Dissemination of information	1	12	30	8	9	60	2.88
Layout	1	11	34	6	8	60	2.87
Intuitive interface	2	12	32	5	10	61	2.78
Navigation and ease of use	2	16	28	7	8	61	2.75
Ability to find what I need	4	17	24	8	8	61	2.68
Skipped	22 out of 83						

17. Would you be interested in receiving additional training and guidelines in familiarization and usage of this MCHSL eSearcher?

Answer	# responses	Percent
Yes	47	57.32
No	32	39.02
Other, please specify	3	3.66
Skipped	1 of 83	1.20
