# Supplementary material for: An evidence-based method for assessing the value of a search tool: a pilot study
Source: J Med Libr Assoc. 2018 Oct 1;106(4):471–6. doi: 10.5195/jmla.2018.287 (PMC6148619; doi:10.5195/jmla.2018.287)
Supplement: Appendix B [file jmla-106-471-s002.pdf]

JMLA Journal of the Medical Library Association

Supplemental content to
J Med Libr Assoc. Oct;106(4):dx.doi.org/10.5195/jmla.2018.287
www.jmla.mlanet.org
© Pearson, Roksandic, Kilanowski 2018

An evidence-based method for assessing the value of a search tool: a pilot study

Donald Stanley Pearson, MBA, MLIS, AHIP; Stevo Roksandic, MBA, MLIS, AHIP; Jill Kilanowski, PhD, RN, APRN, CPNP, FAAN

APPENDIX B

eSearcher study paper response form

<table>
<tr><td colspan="3">Directions:</td><td rowspan="2">MCHSLS Use ONLY:</td></tr>
<tr><td colspan="3">1. Enter your demographic information. This is a confidential, Mount Carmel Health System (MCHSLS) Institutional Review Board approved study.
2. Search prompt: Please locate the most current guideline for the diagnosis and management of asthma in a 55-year-old male patient.
3. Enter search Start Time.
4. When your information search is concluded, enter End Time.
5. Write in the answer to the search prompt; please leave your search screen open on the computer you used.
6. Rank your confidence level (1–7) that your search resulted in information that could be used in an actual patient care setting. If you did not find a suitable answer, make note of that in the comments section, along with the reason. (i.e., got a page/call.) Even if you did not get an answer, please indicate where you searched.
7. Add optional comments</td></tr>
<tr><td>Date: 2/17/2016</td><td>Start time:</td><td>End time:</td><td>Total time:</td></tr>
<tr><td colspan="3">Demographic information (circle ONE):

Male or Female
Age range:
<20 21–25 26–30 31–35 36–40 41–45 46–50 50></td><td rowspan="2">Nursing graduate student</td></tr>
<tr><td colspan="3"></td></tr>
<tr><td>Answer:</td><td colspan="2">Search terms used:
Title:
Database/Internet resource used:
Publication, if applicable:
Date of article/resource:</td><td>Assessment:</td></tr>
<tr><td>Level of confidence (check one)</td><td colspan="2">Not at all confident Extremely confident

1 2 3 4 5 6 7</td><td>Confidence score:</td></tr>
<tr><td colspan="3">Comments:</td><td></td></tr>
</table>
